# Triticum vulgare Extract Modulates Protein-Kinase B and Matrix Metalloproteinases 9 Protein Expression in BV-2 Cells: Bioactivity on Inflammatory Pathway Associated with Molecular Mechanism Wound Healing

**DOI:** 10.1155/2020/2851949

**Published:** 2020-02-27

**Authors:** Niccola Funel, Valentina Dini, Agata Janowska, Barbara Loggini, Massimiliano Minale, Fabrizia Grieco, Salvatore Riccio, Marco Romanelli

**Affiliations:** ^1^Department of Dermatology, University of Pisa, Italy; ^2^Farmaceutici Damor SPA, Napoli, Italy

## Abstract

Matrix metalloproteinases (MMPs) are a large family of ubiquitously expressed zinc-dependent enzymes with proteolitic activities. They are expressed in physiological situations and pathological conditions involving inflammatory processes including epithelial to mesenchymal transition (EMT), neuronal injury, and cancer. There is also evidence that MMPs regulate inflammation in tumor microenvironment, which plays an important role in healing tissue processes. Looking at both inflammatory and neuronal damages, MMP9 is involved in both processes and their modulation seems to be regulated by two proteins: tumor necrosis factor-alpha (TNF-alpha) and interleukin 6 (IL-6). However other important genes are involved in molecular regulation of transcription factors, protein-kinase B (AKT), and p65. In addition, Triticum vulgare extract (TVE) modulated the biological markers associated with inflammatory processes, including p65 protein. While there are no evidence that TVE might be involved in the biological modulation of other inflammatory marker as AKT, we would like to assess whether TVE is able to (1) modulate phosphorylation of AKT (pAKT) as an early marker of inflammatory process in vitro and (2) affect MMP9 protein expression in an in vitro model. The BV-2 cells (microglial of mouse) have been used as an in vitro model to simulate both inflammatory and neuronal injury pathologies. Here, MMP9 seems to be involved in cellular migration through inflammatory marker activation. We simulate an inflammatory preclinical model treating BV-2 cells with lipopolysaccharide (LPS) to induce proinflammatory activation affecting pAKT and p65 proteins. TVE is revealed to restore the native expression of AKT and p65. Additionally, TVE extract modulates also the protein concentration of MMP9. Nevertheless, immunofluorescence confocal analyses revealed that both AKT and MMP9 are regulated together, synchronously. This work seems to demonstrate that two important genes can be used to monitor the beginning of an inflammatory process, AKT and MMP9, in which TVE seems able to modulate their expression of inflammation-associated molecules.

## 1. Introduction

Different processes in human tissue repairing have been associated, in many cases, with cellular damages. The list of phenomena associated with cellular injury includes, but is not limited to, inflammatory responses, necrosis, and mitochondrial dysfunction [1–4].

Looking at the list mentioned above, the big actor is represented by the inflammatory response, in which, the beginning of cellular injury open the way to proinflammatory marker expression inside the damaged cells. However, it is often difficult to understand the *primum movens* of inflammatory molecular process; indeed, many scientists can list the following biomolecular markers, including tumor necrosis factor-alpha (TNF-*α*), interleukin 1beta, (IL-1*β*), interleukin 6 (IL-6), and nitric oxide (NO) as the “fingerprint” of inflammatory process [5, 6]. Nevertheless, nuclear factor kappa beta (NF-*κ*B) is an important transcription factor involved in inflammatory responses, resulting to a major effector of this process [7]. The NF-*κ*B pathway is activated in various clinical injury conditions including both injuries and ischemia in the brain [8]. For these reasons, NF-*κ*B expression and its expression in BV-2 cells are often used as the preclinical model of microglia inflammation [9], in which NF-*κ*B nuclear expression regulates several genes involved in inflammatory; the list includes enzymes, cytokines, receptors, and cell adhesion molecules [10]. So far, to investigate in vitro this process, many scientists have used the BV-2 cell cultures, derived from mouse microglia. In fact, stimulation by lipopolysaccharide (LPS) in BV-2 cells affects protein modulation of other messengers of mitogen activation such as glycogen synthase kinase (GSK-3*β*) protein and phosphoprotein-kinase B (PKB or pAKT) [11]. Additionally, the molecular scenario of inflammation includes important proteins/enzymes. There are other important evidences indicating the matrix metalloproteinases (MMPs) as major actors in ischemia/reperfusion-induced brain injury [12, 13], in a dependent manner via NF-*κ*B action also. In particular, MMP9 was upregulated following cerebral ischemia in experimental animal models [14]. Further, TNF-*α* and IL-1*β* have been reported to induce the production of MMPs [15–17]. Looking at this complex network of molecules involved in inflammation models seems to be useful to investigate the relationship between the early marker of inflammation and the end effector as MMPs. The possibility to study new therapeutic approaches affecting proinflammatory response targeting the beginning driving genes (i.e., AKT) and the final effectors (MMPs) seems to be a promising clinical treatment for all pathologies in which the inflammatory process drives the pathological behaviors [18]. Triticum vulgare extract (TVE) demonstrated to modulate several proinflammatory messengers in BV-2 models, but its efficacy is not well demonstrated looking at the expression of AKT and MMP9 in the model mentioned above. However, other important studies demonstrated that TVE is commonly used for the treatment of different pathological conditions of the skin, including ulcers, burns, and dystrophic diseases [5], in which reepithelization or tissue regeneration processes are associated with the inflammatory process. In fact, it has been reported that the active component of Fitostimoline products (TVE) stimulate the acceleration of tissue repairing, chemotaxis and the maturation of fibrotic cells, and healing process [19–22]. Indeed, looking at the whole scenario around the TVE activities, we are asking ourself whether TVE could be assimilated inside the category of a pharmaceutical compound labeled as “bioactive compound.” One of the definition used in order to establish a definition of bioactive compound said: “*Bioactive compounds in plants are compounds produced by plants having pharmacological or toxicological effects in man and animals*” [23]. Nevertheless, biological molecules induce pharmacological (good) or toxicological (bad) effects when ingested at high dosages (e.g., vitamins and minerals). Often, the bioactive compounds in plants are derived as secondary compound. Indeed, we would like to coin a definition of bioactive compounds as follows: “plant-derived secondary metabolites exploiting pharmacological or toxicological effects in man and animals.” The goal of the present work was to establish whether TVE might be categorized as a bioactive compound, modulating AKT and MMP9 protein expression in an in vitro system, in relation to the major actor of inflammation as NF-*κ*B.

## 2. Materials and Methods

### 2.1. Triticum vulgare Extract (TVE)

Triticum vulgare, the binomial scientific name of a plant of Graminaceae family, is the commonly known wheat plant. It is grown under controlled conditions in the laboratory of Farmaceutici Damor, Naples, Italy. The voucher specimen is DF/237/2014 and it is deposited in the herbarium of the Medical Botany Chain of University of Salerno, Italy. The commercially available seeds are purchased from Consorzio Agrario Lombardo Veneto from Northern Italy. The batch number for the seeds used for the present paper was 12/001-B10148/201/04. Triticum vulgare extract (TVE-DAMOR) is a specific aqueous extract of Triticum vulgare, obtained by a complex and specific process as already described [24]. It was a gift of Farmaceutici Damor (Naples, Italy).

### 2.2. Cell Line

The immortalized murine BV-2 cell line (ICLC ATL03001, Interlab Cell Line Collection, Banca Biologica e Cell Factory, Italy) was cultured in Dulbecco's Modified Eagle's Medium (DMEM, Invitrogen) supplemented with 10% fetal bovine serum (FBS), 1% penicillin-streptomycin (Invitrogen), and 1% glutamine (Invitrogen). Cultures were grown at 37°C in 5% CO_2_ until 80% confluence. In order to perform the treatments and analyses, cells were split when they reached confluence using trypsin/EDTA solution in PBS. We used two different modalities of seeding according to the different molecular determination investigated below.

#### 2.2.1. For MMP9 Determination by ELISA Test

BV-2 mouse microglial cells were seeded in 12-well plates, in order to obtain three different experiments for each concentration of TVE with and without LPS. The mediums were harvested for analyses as described in the following section.

#### 2.2.2. To Analyze AKT, MMP9, and p65 Protein Expressions by Confocal Immunofluorescence

BV-2 mouse microglial cells were seeded in 8-well chamber slides (CS) (Lab-Tek1 Chamber Slide™ system, Nalge Nunc International, Naperville, IL, USA), putting in 5000 cells/well in 650 *μ*L final volume. CS were prepared in order to obtain three different experiments in triplicate. After treatments, cells were fixed directly on the slides by a 70% solution of ethanol solution for 10 minutes and the chamber slide wells were removed by a mechanical key following manufacturer's instructions. The immunofluorescence (IF) for AKT, MMP9, and p65 subunits was performed as described in the immunofluorescence section.

### 2.3. In Vitro Treatments

BV-2 cells and cells were seeded in 12-wells plate. Cells were treated with LPS and TVE. BV-2 cell lines were exposed to the following concentration: 5%, 10%, and 20% of TVE. Twelve hours after TVE prestimulation, cells were incubated with the lipopolysaccharide- (LPS, Sigma) simulating inflammatory stimulus (100 ng/mL, 24 hours). We performed a total of six chamber slides in order to perform three different concentrations of TVE with and without LPS.

**Figure 1:**
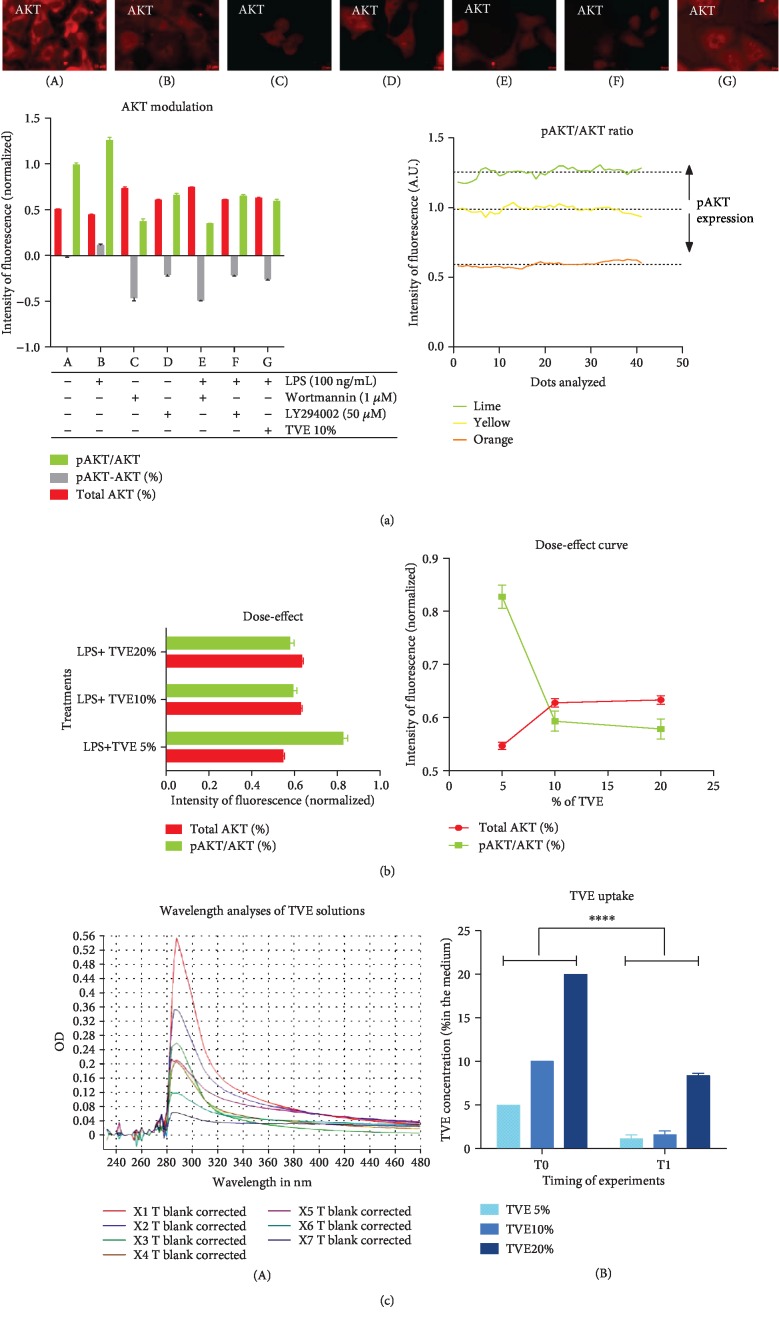
AKT functional analyses. The analyses of pAKT and total AKT (by arbitrary units A.U.) were revealed through confocal immunofluorescence in BV-2 cells. (a) IF of treated cells. (A) Controls. (B) LPS. (C) Wortmannin. (D) LY294002. (E) Wortmannin+LPS. (F) LY294002+LPS. (G) TVE+LPS. Graphs of fluorescence intensity and pAKT modulation according to the IF spectra reported in Figure 1. (b) Dose-effect evaluation of pAKT and total AKT expressions. (c) Uptake evaluation by spectrophotometric analyses. (A) OD of 7 TVE concentrations: X1 = 100%, X2 = 50%, X3 = 40%, X4 = 30%, X5 = 20%, X6 = 10%, and *X*7 = 5%. (B) Bar graph reporting the difference of TVE concentrations in the medium at the beginning (T0) and the end (T1) of experiments.

In addition, we treated the BV-2 cells with two specific inhibitors of pAKT, wortmannin (1 *μ*M; #9951, EuroClone, Milan, Italy) and LY294002 (50 *μ*M; #9901, Euroclone, Milan, Italy). We treated BV-2 cells using these two inhibitors, separately, and each of them in combination with LPS, in order to verify a functional action with respect to pAKT status. Control groups were obtained to avoid any compound. Finally, we evaluated seven different treatments, as follows: (A) Controls, (B) LPS, (C) Wortmannin, (D) LY294002, (E) Wortmannin+LPS, (F) LY294002+LPS, and (G) TVE+LPS (Figure 1(a)).

### 2.4. Determination of MMP9 Protein Expression by ELISA

MMP9 protein concentration was assessed as supernatants of BV-2 cell cultures with a commercially available kit (cytokine, R&D, Bio-Techne, Milan). Cellular mediums were centrifuged at 4000 rpm for 5 min. Levels of MMP9 were measured by the enzyme-linked immunoassay (ELISA) according to the manufacturer's instructions. In agreement with the manufacturer's instructions, all experiments were performed in triplicate and the calibration curve was assessed. The acquisition of values and the calculation of their concentration were performed by multireader instruments and its software (SPECTROstar Nano, EuroClone, Milan, Italy).

### 2.5. Determination of Nuclear Concentration of p65 Protein by Immunofluorescence

The quantification of NF-*κ*B p65 subunit in BV-2 cells was performed by confocal immunofluorescence methodology. The plastic covers of chamber slides were removed at the end of pharmacological treatment, by a key dedicated. The slides were fixed by ethanol (70%, 10 min) and then rinsed in phosphate buffer saline (PBS 1x for 10 min). In order to detect p65 subunit, we used a polyclonal antibody, (1 : 100; 1 h at RT; NF-*κ*B p65 (D14E12) XP1 Rabbit mAb #8242, Cell Signaling Technologies, Leiden, Netherlands). After washing, the fluorescent secondary antibody was applied (1 : 50; 30 min at RT in darkness; Anti-rabbit IgG Fab2 Alexa-Flour 488, #4412S, Molecular Probes, Cell Signaling Technologies, Leiden, Netherlands). Nuclei counterstaining was performed using a special fluorescence antifade containing DAPI (ProLong1 Gold Antifade Reagent with DAPI #8961, Molecular Probes Cell Signaling Technologies, Leiden, Netherlands). Samples were stored at 4°C until observation. The visualization, nuclear migration, and quantification of NF-*κ*B p65 subunit were performed using a confocal microscope (AXIO vert 200, Zeiss, Wetzlar, Germany) and its dedicated software for imaging acquisition and digital imaging process (AXIOvision version 4.2.3.1, Zeiss, Wetzlar, Germany). The images were acquired at 40x magnification. Five different images (DAPI, green, merge (M), bright field (BF), and BF+M) were acquired for each field and are reported in the corresponding figure.

To quantify the signal of each color channel (blue and green), we have to draw a vector on merged images in order to obtain a graph reporting the intensity of IF, looking at IF signals in both the cytoplasm and nuclei. The vector analyzed 40 different points/cell, across the nucleus and cytoplasm. The length of the vector was equal to 6 *μ*m. The intensity of aqua spectrum was calculated by the ratio (IF R) between IF values obtained by the blue channel out of those obtained by the green channel. Nuclear protein expression of p65 was associated with the presence of the aqua color. The range of aqua spectrum ranged as follows: 1.00 < IF R < 1.40. Indeed, we overlap the BF images to verify the location of the aqua color inside the nuclei [5].

### 2.6. Determination of Cytosol Concentration of AKT Proteins by Immunofluorescence

**Figure 2:**
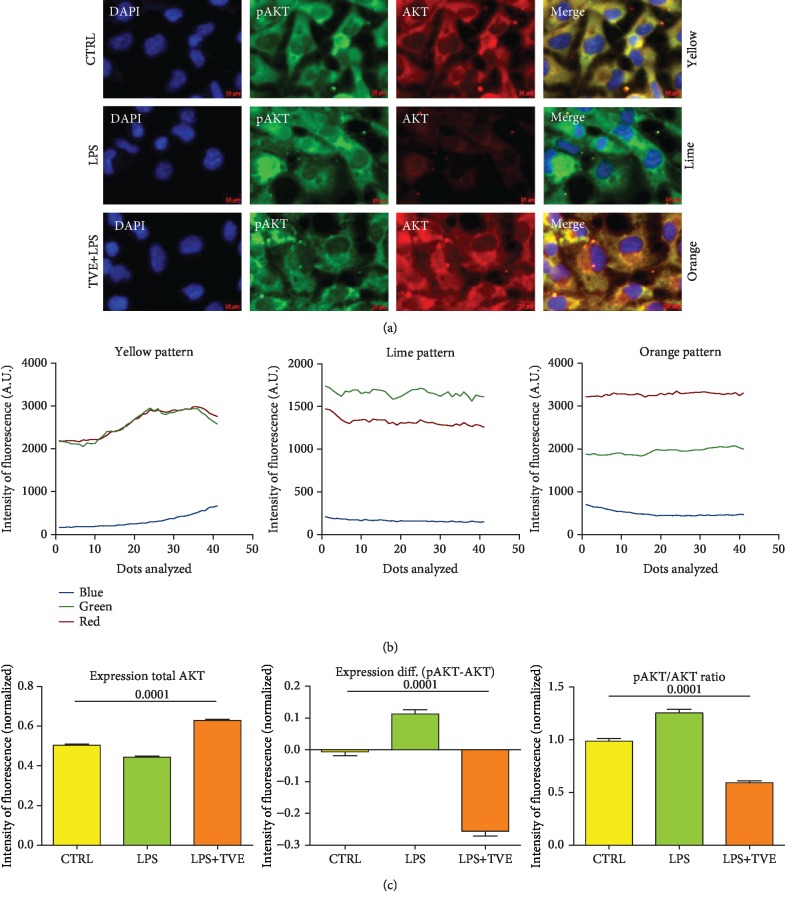
AKT protein modulation. (a) Confocal immunofluorescence representation of AKT protein modulation (total and phosphorylated forms) in Controls and the TVE- and TVE+LPS-treated BV-2 cells. (b) The overlapping spectra (blue: DAPI; green: pAKT; and red: total AKT) highlighted three major color spectra: yellow (Y) when pAKT≅total AKT, lime (L) when pAKT > total AKT, and orange (O) when pAKT < total AKT. (c) LPS treatment upregulated pAKT form (L), wherever TVE restored the AKT status increasing the unphosphorylated form of protein (O). TVE treatment affected the pAKT/AKT ratio as well.

We repeated the same procedure as described in the previous section to detect both forms of AKT, protein phosphorylated (pAKT) and unphosphorylated (total AKT). Total AKT was detected by monoclonal antibody, (1 : 100; 1 h at RT; AKT pan (40D4) XP1 Mouse mAb #2920, CST, Leiden, Netherlands), while phospho-AKT was detected by polyclonal antibody (1 : 100; 1 h at RT; pAKT (D9E) Rabbit mAb #4060, CST, Leiden, Netherlands). After washing, two fluorescent secondary antibody was applied (1 : 50; 30 min at RT in darkness; Anti-mouse IgG Fab2 Alexa-Flour 535, #4412S (Red) and Anti-rabbit IgG Fab2 Alexa-Flour 488, #4412S (Green), Molecular Probes, CST, Leiden, Netherlands). Nuclei counterstaining was performed using a special fluorescence antifade containing DAPI (ProLong1 Gold Antifade Reagent with DAPI #8961, Molecular Probes CST, Leiden, Netherlands). In the AKT experiments, the vector analyzed 40 different points/cell, inside the cytoplasm. The length of the vector was the same with that used for the p65 protocol (6 *μ*m). Combining red and green spectra associated with AKT and pAKT, respectively, we obtained three different additional spectra calculated by the ratio (IF R) between green and red (green/red). The yellow spectra (Y) were observed when IF R = 1. While, when IF *R* > 1, we observed a typical lime (L) color; otherwise, for IF R < 1, we observed a typical orange (O) color. We overlapped the BF images, to verify the location of the yellow, lime, and orange colors inside the cytoplasms of BV-2 cells (Figures 1 and 2).

### 2.7. TVE Uptake and pAKT Modulation Curve

The uptake of TVE was calculated by a multireader instrument and its software (SPECTROstar Nano, EuroClone, Milan, Italy), using the Lambert-Beer equation. We evaluated the concentration of TVE using the optical density (OD) values obtained at 293 nm wavelength; this wavelength represents the maximum OD of TVE. The calibration curve was obtained by analyzing 7 different concentrations of TVE in the same medium used for the experiments. The uptake of TVE was evaluated comparing the TVE concentration and the beginning (T0) and the end (T1) of treatment. We assumed that differences of concentration (T0-T1) was associated with the TVE uptake by BV-2 cells. We evaluated the TVE uptake in BV-2 cells after with 5%, 10%, and 20% treatments of TVE in combination with LPS, respectively. The cells of the same experiments were assessed for both pAKT and AKT immunofluorescence, as described above. The relation between TVE uptake and pAKT/AKT ratio was evaluated to find a dose-response curve.

### 2.8. Determination of Cytosol Concentration of MMP9 Protein by Immunofluorescence

The MMP9 IF detection was performed in the same way following the procedure used for p65 and AKT markers. MMP9 protein was detected by polyclonal antibody (1 : 100; 1 h at RT; MMP9 (D603H) XP rabbit mAb #13667, CST, Leiden, Netherlands). After washing, fluorescent secondary antibody was applied (1 : 50; 30 min at RT in darkness; Anti-rabbit IgG Fab2 Alexa-Flour 535, #4412S (Red) Molecular Probes, CST, Leiden, The Netherlands). Nuclei counterstaining was performed as reported above. To demonstrate both MMP9 and p65 in the same experiments, we performed simultaneously IF for these two markers, red for MMP9 and green for p65. The goal of these experiments was to find cytoplasmic spectra Y, associated with MMP9 and p65 expressions and aqua spectra associated with p65 nuclear localization. The quantification of fluorescence intensity was analyzed for each experiment and reported by the graphs.

### 2.9. *In Silico* Analyses of Inflammation Markers

In order to evaluate the interaction between the “fingerprint” markers of inflammation, p65, and MMP9, we evaluated 13 articles in which the researchers studied the protein expression of the genes mentioned above, through ELISA, Western Blot, and/or immunofluorescence. In each article, we evaluated as proinflammatory agents (PIA) the molecules or compounds capable of increasing the protein expression of markers. While we considered anti-inflammatory agents (AIA) the treatments that were able to restore the same protein expression promoted by PIA. Looking at the results for each article, we evaluated the fold-charge of each protein and each treatment compared to their controls.

**Figure 3:**
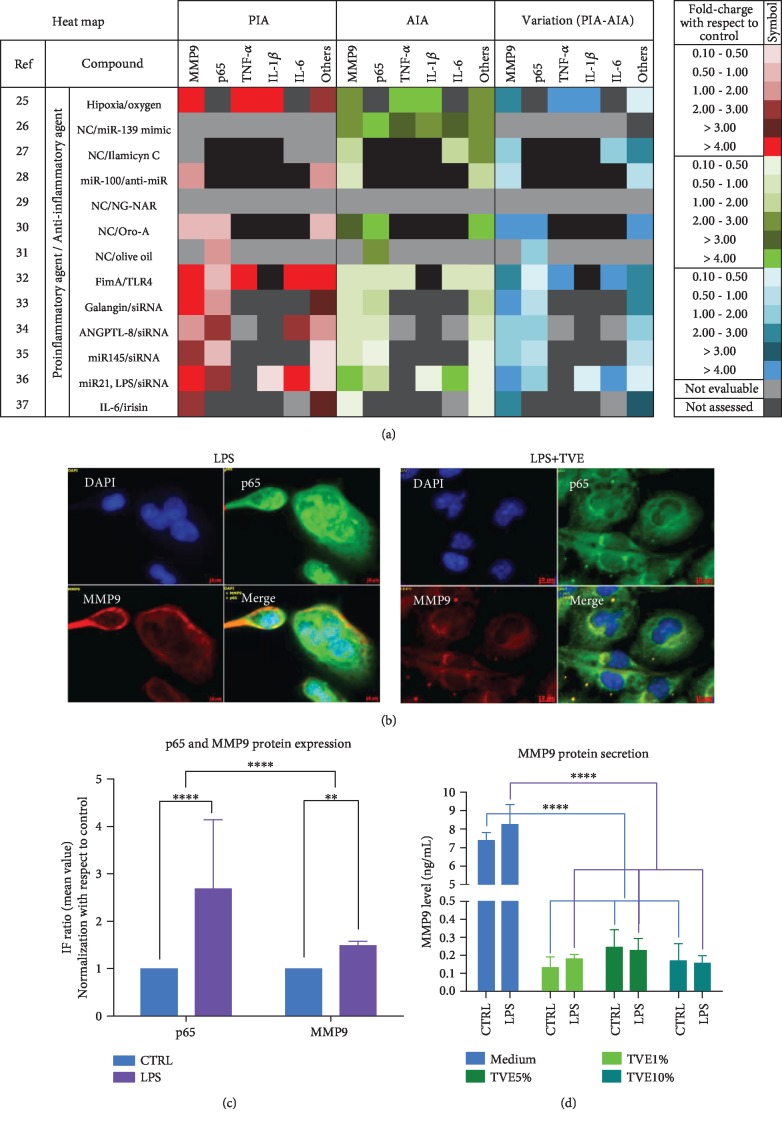
MMP9 protein analyses. (a) Heat-map of *in silico* analyses of protein expression of major proteins involved in inflammatory molecular pathway. The values of fold-charge expression were reported in the right side of the figure. PIA: proinflammatory agent; IAI: anti-inflammatory Agent. (b) Coexpression of p65 and MMP9 in BV-2 cells after treatments. Double immunofluorescence for p65 (green) and MMP9 (red) in BV-2 cells after LPS treatment and control. Upregulation of MMP9 (yellow spectra, cytoplasm) and p65 (Aqua spectra, nuclear). p65 regulation after treatments. Upregulation (by LPS) and downregulation (by TVE) of p65 in the nuclei of BV-2 cells. (c) Quantification of p65 and MMP9 protein expressions. (d) Analyses of MMP9 secretion obtained by ELISA test. ns: not significant differences; ^∗^*p* < 0.05; ^∗∗^*p* < 0.001; ^∗∗∗^*p* < 0.001; ^∗∗∗∗^*p* < 0.0001.

To have a quick interpretation of the results, we have elaborated a heat map to visualize PIA, AIA, the markers studied, and their modifications, for each article. The final visualization of the heat map was obtained based on the intensity of the fold-charge evaluated (Figure 3). The articles that we have been using for this part of our work covered the following topics: brain injury [25, 26], inflammatory mechanism [27–32], and cancer [33–37]. The specifications of papers included in these analyses are reported in Table 1.

### 2.10. Statistical Analysis

Experiments were performed at least three times and the data are expressed as the mean SEM of the values obtained in three separate experiments. Statistical comparisons between controls and treated groups were performed by one-way analysis of variance (ANOVA). The *p* < 0.05 values were considered significant.

## 3. Results and Discussion

**Figure 4:**
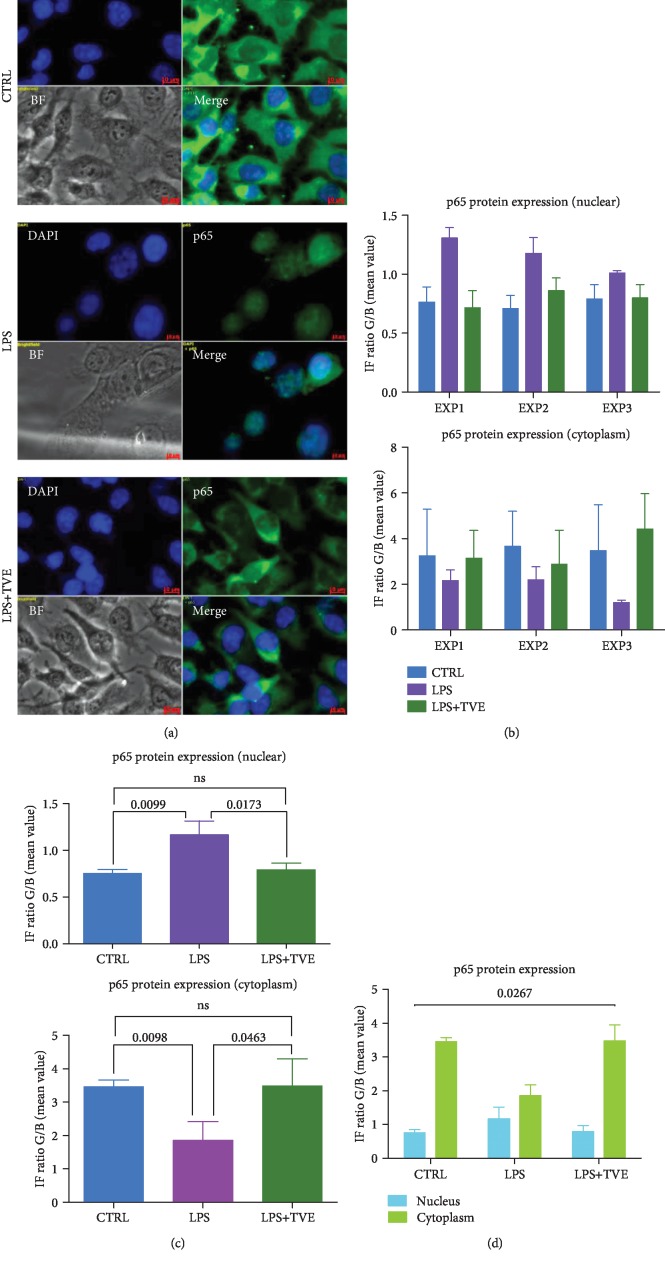
p65 protein analyses. (a) IF on BV-2 cells. (b) Raw data according to both nuclear and cytoplasmic expressions by three different experiments. (c) Quantification p65 protein expression in BV-2. (d) Distribution and localization of p65 in BV-2 cells after treatments. Significant *p* values were reported inside the graph. ns: not significant differences.

Looking at the literature regarding the in vitro model simulating the molecular inflammatory mechanism, BV-2 cells are used for this aim, in several fields [5, 9, 11, 18]. Previously, we demonstrated that this cell culture system modulated inflammatory mediators, such as tumor necrosis factor-alpha (TNF-*α*), interleukin 1beta (IL-1*β*), nitric oxide (NO), and nuclear factor kappa beta (NF-*κ*B), after LPS stimuli [5]. In this study, we demonstrated that our experiments reflexed the cellular behaviors reported by other studies including mechanisms of inflammation [27–32]. In particular, we highlighted the induction of proinflammatory messengers mentioned above (TNF-*α*, IL-1*β*, NO, and NF-*κ*B) [5]. Nevertheless, addition of Triticum vulgare extract (TVE), in combination with LPS showed an anti-inflammatory action on specific markers of inflammation, restoring cytoplasmic levels of p65 and reducing its nuclear expression [5]; this type of mechanism has been reported by other works, in which the p65 restoration (reduction of nuclear staining of p65) is well considered an anti-inflammatory signal [11, 27–32]. However, no other markers have been associated with “early inflammatory molecule” concept, except the four markers mentioned above. Furthermore, this study not only showed the restoration of p65 in BV-2 cells (Figure 4) but also demonstrated the induction of AKT marker, increasing its phosphorylated isoform after LPS, as a simulation of inflammatory process. While the LPS+TVE combination reduced the ratio between pAKT/AKT (Figure 2), indeed, the comparison with respect to other two chemical pAKT inhibitors revealed a decrease of its phosphorylated status (Figure 1(a)). Furthermore, TVE seems to affect pAKT status through a dose-effect manner (Figure 1(b)), where the uptake of TVE concentration showed a significant effect at 5% and 10%, but not at 20% (Figure 1(b)). Indeed, looking at the ratio of pAKT/AKT, TVE treatments seem to play a role as an anti-inflammatory modulator, affecting pAKT expression significantly (Figure 2). This phenotypical description of AKT protein has been reported by other researchers [11]. In particular, this work described also other two genes, GSK-3*β* and Notch-1, involved in the inflammatory model of microglia [11] and in molecular mechanisms of brain cancer [38], where GSK-3*β* seems to modulate the expression of NF-*κ*B [39]. The activation of NF-*κ*B is mediated by phosphorylation and subsequent degradation of inhibitor of *κ*B (I*κ*B) and nuclear migration of p65 subunit [5]. This process subsequently leads to translocation of free NF-*κ*B protein (p65) to the nucleus where it promotes the expression of proinflammatory genes such as the proinflammatory cytokines (TNF-*α*, IL-6, and IL-1*β*), cyclooxygenase-2 (COX-2), and inducible nitric oxide synthase (iNOS) [39, 40]. So far, it is known that NF-*κ*B plays a pivot role inside the program of transcription activation in different types of pathologies [5, 11, 16, 38]. Nevertheless, other researchers reported that NF-*κ*B is also involved in molecular pathway of neuronal apoptosis [9] and malaria infection [18]. In these two papers, the scientific evidences reported that NF-*κ*B was associated with MMP9 protein modulation also [9, 18]. Our result demonstrated that not only the modulation of p65 in BV-2 cells (Figure 4) but also the modulation of p65 is concurrently associated with MMP9 expression (Figure 3). In our in vitro model, the upregulation of p65 nuclear expression after LPS treatment was reverted in the presence of 10% TVE, strongly supporting the anti-inflammatory action of TVE. It is necessary to underline that our quantitative methodology (confocal IF) demonstrated the impact of p65 in our model [5], affecting both phenotypical expression and secretion of MMP9 protein in a statistically significant manner in BV-2 cells (Figure 3). Indeed, the comparison with two other pAKT chemical inhibitors confirmed a reduction in its phosphorylated state (Figure 1(a)). Furthermore, our results appear to be strongly supported by our in silico analyses, in which 8 out of 13 articles studied p65 and MMP9 together as inflammatory markers. Here, 87.5% (7/8) of these works demonstrated a concomitant upregulation of PIA expression of p65 and MMP9 proteins and their restoration with AIA treatments [26, 30–33, 35, 36]. Furthermore, our in silico analyses also revealed that pAKT, MMP9, and p65 were considered together as inflammatory markers and were underregulated by Galangin, a possible inflammatory modulator candidate in brain diseases [26]. With regard to the possible mechanism of this effect, we are in agreement with other studies, in which the concomitant modulation of NF-*κ*B and MMP9 has been demonstrated [11, 18]. Looking at the previous and past work around the molecules involved in the “early inflammatory” process, we highlighted that mediators, such as IL-1, TNF-*α*, IL-6, NO, and PGE2, facilitate the process of tissue repair and remodeling as a result of the damage suffered. It is known that the specific preparation of TVE acts on the fibroblast activating and promoting the process of tissue repair and healing [19–21], exerting an anti-inflammatory action that allows the way of healing to go from the inflammatory process to the regenerative one. Finally, the last but not the least aspect, we can assume that TVE extract works as a bioactive compound, looking in its noncytotoxic effects and the definition of bioactive compounds derived from the plants [23].

## 4. Conclusions

TVE noncytotoxic properties are able to modulate the protein expression of AKT, p65, and MMP9 involved also in inflammation-associated pathology, including wound lesions, working as bioactive a compound inside healing processes.

## Figures and Tables

**Table 1:** In silico analyses reported the characteristics of papers which compare the expression of the MMP9 and p65 proteins included in these analyses. The results were reported in Figure 3(a) through a heat map.

ID paper	Reference	Molecules analyzed		Proinflammatory action with respect to control	Anti-inflammatory action with respect to control	Markers	Pathology	Compound
		RNAs	Proteins					
1	25	-	X	Y	Y	MMP9, TNF-*α*, IL-1*β*, other	Brain injury	Oxygen
2	26	X	X	Y	Y	MMP9, TNF-*α*, IL-1*β*, IL-6, p65	Colon cancer	miR-139
3	27	-	X	N	Y	MMP9, IL-6, other	Brest cancer	Ilamycin C
4	28	-	X	Y	Y	MMP9, IL-1*β*	Inflammatory mechanism	miR-100
5	29	X	-	N	Y	MMP9, IL-1*β*, IL-6	Inflammatory mechanism	NG-NAR
6	30	X	X	Y	N	MMP9, p65, other	Neck cancer	Oro-A
7	31	X	-	N	Y	p65	Inflammatory mechanism	Olive oil
8	32	X	X	Y	Y	MMP9, TNF-*α*, IL-6, p65, other	Inflammatory mechanism	FimA
9	33	X	X	Y	Y	MMP9, AKT, p65, other	Brain injury	Galangin
10	34	X	X	Y	Y	MMP9, TNF-*α*, IL-6, p65, other	Inflammatory mechanism	ANGPTL-8
11	35	X	X	Y	Y	MMP9, p65, other	Inflammatory mechanism	miR-145
12	36	X	X	Y	Y	MMP9, IL-6, p65, other	Neck cancer	miR-21
13	37	X	X	Y	Y	MMP9, IL-6, p65, other	Osteosarcoma	IL-6

X:done; -: not done; Y: yes; N: no.

## Data Availability

1. All data concerning original pictures, figures, and graphs used to support the findings of this study are included within the article. 2. The original files (raw data) concerning pictures, figures, graphs, and any electronic file generating data used to support the findings of this study are available from the corresponding author upon request.
